# Immunogenicity and protective potential of a mucosal protein-only vaccine candidate for tuberculosis

**DOI:** 10.3389/fimmu.2026.1810847

**Published:** 2026-06-23

**Authors:** AC Tran, MY Kim, EJ Vergara, MJ Paul, D Burnsall, M Das, JE Pearl, AM Cooper, Rajko Reljic

**Affiliations:** 1 Institute for Infection and Immunity, School of Health and Medical Sciences, City St. George’s University of London, London, United Kingdom; 2 Department of Molecular Biology and the Institute for Molecular Biology and Genetics, Jeonbuk National University, Jeonju, Republic of Korea; 3 Department of Infection, Immunity and Inflammation, University of Leicester, Leicester, United Kingdom

**Keywords:** infection, lung, mice, mucosal, TB vaccine

## Abstract

Despite the widespread use of the BCG vaccine, tuberculosis remains a leading global health threat. The primary limitation of BCG lies in its failure to prevent pulmonary TB in adults, largely due to its systemic administration route which fails to induce robust mucosal immunity at the site of primary infection. This study evaluates a novel mucosal vaccine platform, TB-PCF, incorporating *Mycobacterium tuberculosis* antigens ESAT6 and CFP10 fused with Cholera Toxin B subunit (CTB) and an IgG-Fc domain to enhance polymerisation and antigen uptake by antigen-presenting cells. Following systemic priming and mucosal boosting in mice, TB-PCF vaccine elicited significant antigen-specific antibody responses in serum and bronchoalveolar lavage fluid, as well as polyfunctional systemic Th1 and Th17 responses, characterised by elevated IFN-γ and IL-17. Notably, splenocytes from vaccinated mice exhibited significant bacterial killing in a modified mycobacterial growth inhibition assay (MGIA), trending higher than BCG. However, in the subsequent *in vivo* challenge test, only the BCG-vaccinated group achieved a statistically significant reduction in lung bacterial burden. While the failure to translate *in vitro* bacterial killing into *in vivo* protection suggests that the ESAT6-CFP10 antigen duo may lack the necessary antigenic breadth for full protection, we propose that the TB-PCF platform is a promising, new tool for future screening of diverse antigen combinations to overcome current roadblocks in mucosal TB vaccination.

## Introduction

Tuberculosis (TB) remains one of the world’s deadliest infectious diseases, with 10.7 million new cases in 2024 (1), particularly in regions such as sub-Saharan Africa and Southeast Asia. Current treatment options involve long-term antibiotic regimens, but the emergence of drug resistance highlights the necessity for more effective vaccines that can prevent infection and reduce transmission. The only licensed vaccine against TB is the BCG (Bacillus-Calmette Guerin) vaccine; however, its efficacy is variable, particularly against adult pulmonary TB (2). Being a live vaccine, BCG is also contraindicated for HIV positive or immunocompromised individuals, and there is an urgent need for improved vaccination strategies (3). The development of next-generation TB vaccines that can elicit robust systemic and mucosal immune responses is critical to achieving global TB control, while scalability of production and simplified storage requirements can ensure global health equity.

One of the main challenges for developing protein-based vaccines is the paucity of available, human-applicable adjuvants. While protein antigens are poorly immunogenic and require adjuvants to elicit protective, long-lasting immunity and enable dose-sparing, adjuvant development remains a critical “bottleneck.” For decades, alum was the sole licensed option (4). Since the 1990s, only a few alternatives, such as MF59, AS0, CpG1018, and MatrixM, have reached clinical use (5). This slow pace of innovation stems from the complex challenge of balancing enhanced immunogenicity with safety. While necessary for potency, adjuvants carry inherent risks of toxicity, ranging from mild local irritation to rare, serious systemic adverse events. Mucosal adjuvants face even stricter safety requirements than systemic ones due to their proximity to the central nervous system and delicate epithelial barriers. Intranasal delivery, for instance, poses risks of retrograde transport to the brain via the olfactory bulb, a concern highlighted by the withdrawal of an LTK63-adjuvanted intranasal HIV vaccine linked to Bell’s palsy (6). Additionally, mucosal surfaces are prone to local inflammation and hyper-responsiveness, which can damage tissue integrity or trigger allergies. Consequently, adjuvants must maintain a narrow balance: being potent enough to break mucosal tolerance without inducing neurotoxicity or chronic inflammation.

To reduce reliance on exogenous adjuvants and simplify vaccine formulations, our research has focused on the molecular engineering and testing of vaccine candidates that autonomously generate their own adjuvanticity. These candidates are based on Immunoglobulin G (IgG) Fc fragments fused with target antigens, engineered to polymerise and form immune complexes that enhance antigen presentation by dendritic cells and other antigen-presenting cells (APCs) (7, 8). Previous iterations of these Fc-fusion proteins have demonstrated significant vaccine potential against Dengue (9–13) and SARS-CoV2 (14).

In the context of TB, we have previously developed a novel, protein-based vaccine platform that derives its adjuvanticity from fusing the TB antigen with the IgG-Fc fragment, which carries the IgM-tail piece responsible for hexamerisation, leading to polymeric immune complexes (15). This platform, known as the Polymeric Immunoglobulin Scaffold (PIGS), has shown promise in preclinical models by inducing strong humoral and cellular immune responses, including memory and effector T cells. However, this approach has limitations in inducing mucosal immunity, which is crucial for TB control given the pathogen’s primary site of infection.

To address these limitations, we have developed a new vaccine platform that combines the immune-enhancing properties of the Fc-fusion protein with a molecular adjuvant, the cholera toxin non-toxic B subunit (CTB), known for its ability to recruit APCs to mucosal sites. This new platform, termed PCF (Platform CTB-Fc), integrates three molecular components: CTB, the TB antigens ESAT6 and CFP10 (in the form of ESAT6-CFP10 fusion protein), and the IgG Fc fragment, within a single polypeptide. The CTB component acts as a mucosal immune modulator, facilitating the influx and activation of APCs (12), while the polymeric Fc fragment enhances antigen uptake and presentation (13). The PCF platform forms pentamers through CTB (16), delivering multiple copies of the antigen to APCs, thereby amplifying the immune response.

In this study, we present evidence demonstrating the robust immunogenic properties of this novel self-adjuvanting vaccine delivery system for TB *in vitro* and *in vivo*, and we show that it can induce strong systemic and mucosal immunity.

## Materials and methods

### Production and purification of recombinant ESAT6-CFP10 antigen

To express ESAT6-CFP10 fusion protein, the gene for ESAT6 (UniProt, P9WNK7) linked with CFP10 (UniProt, P9WNK5) of *Mycobacterium tuberculosis* (Mtb) (strain ATCC 25618/H37Rv) was synthesised by Cosmogenetech (South Korea), with penta-glycine used as a linker between ESAT6 and CFP10. The ESAT6-CFP10 gene was amplified from carrier plasmid DNA using PCR with Phusion polymerase (New England Biolabs), and the resulting product was gel-extracted. The ESAT6-CFP10 insert was digested with BamHI-HF and XbaI, ligated into a pCOLD II vector, and transformed into BL21 *E. coli* cells (ThermoFisher Scientific). Colonies were screened by PCR, and positive clones were expanded in LB medium with carbenicillin. Protein expression was induced with 10 mM IPTG at 15 °C, and cells were lysed by sonication. The expressed ESAT6-CFP10 protein (23.2 kDa) was confirmed by SDS-PAGE and Western blot analysis. The protein was subsequently purified, dialysed against 50mM ammonium bicarbonate buffer, and endotoxin was removed using polymyxin B agarose (Sigma-Aldrich). The final purified protein was concentrated using a 3.5kDa cut-off centrifugal concentrator (Amicon) and stored at 4 °C.

### Construction of TB-PCF vaccine delivery platform and expression in plants

The *Nicotiana benthamiana* plant expression system was used to express TB-PCF. The gene sequences of ESAT6 and CFP10 were codon optimised for plant expression (Cosmogenetech (South Korea) and *BamHI* and *SpeI* restriction enzyme sites were added at the N and C-terminus, respectively. The ESAT6-CFP10 was then digested with *BamHI* and *SpeI* and subcloned into the pTRAk plant expression vector, containing PCF backbone as described in a previous study (13). This plant expression vector was then electroporated into *Agrobacterium tumefaciens* strain GV3101 containing the pMP90RK helper plasmid. Subsequently, a positive clone was identified through colony PCR and utilised for transient expression.

*Agrobacterium tumefaciens* cultures transformed with the expression vector were initiated in 15 mL of Luria-Bertani (LB) medium supplemented with 50 µg/mL kanamycin and rifampicin and incubated at 28 °C for 24 h on a shaking platform. The cultures were then scaled up to 200 mL of LB medium containing the same antibiotics and incubated for an additional 6 h under identical conditions. Following two rounds of washing with infiltration buffer (10 mM MES, 10 mM MgCl_2_, pH 5.6), the bacterial cells were resuspended in infiltration buffer to a final optical density of 1 at 600 nm (OD600). The cultures were then introduced into a vacuum chamber containing whole *N. benthamiana* plants. A 3-min vacuum treatment was applied, followed by equilibration to atmospheric pressure, which facilitated leaf infiltration. Post-infiltration, the plants were grown under controlled conditions (180 µE/m²s light intensity, 16-h photoperiod, 26 °C, and 50–80% relative humidity) for 5 days prior to harvesting. Infiltrated leaves were homogenised in a blender with an additional 3-fold volume of PBS supplemented with 0.01% (w/v) Triton X-100, relative to the leaf weight. The resulting homogenates were centrifuged at 12,000 rcf to pellet particulates and the supernatants collected through Miracloth (Merck Millipore) to remove coarse debris. Subsequently, the clarified supernatants were filter sterilised using 0.22 µm PES filters in preparation for affinity chromatography purification.

### Purification and analysis of TB-PCF

Protein purifications using Protein A agarose (Sigma), concentration and fractionation by size-exclusion chromatography (SEC) were performed as previously described (14). The protein concentration was measured by absorbance at 280 nm using the NanoDrop™ 2000/2000c Spectrophotometer (ThermoFisher Scientific). The final concentration was calculated using an extinction coefficient factor of 1.107, based on the ProtParam tool in Expasy (https://web.expasy.org/protparam/) for *in vitro* characterisation. To perform electrophoresis and Western blotting, precast gels from Life Technologies were used. SDS-PAGE was performed under reducing (R) or nonreducing (NR) conditions, using 4%–12% BIS/TRIS gels. Following electrophoresis, the gels were subjected to Coomassie staining or Western blot analysis. The protein transferred membranes were incubated in 5% non-fat dry milk protein solution in PBS for all incubation and washing steps. Specific anti-ESAT6 antibodies (Creative Biolabs, 1:2000 dilution), followed by a 1:2500 dilution of peroxidase-conjugated antiserum specific to the light chain of mouse IgG (Jackson ImmunoResearch) were used to detect TB-PCF and ESAT6-CFP10 alone. Finally, the blots were washed and developed using the ECL Plus Western blotting detection system from GE Healthcare and imaged using the G:Box (Syngene).

### Characterisation of TB-PCF by DLS and SEC analysis

Particle size analysis of TB-PCF was conducted using dynamic light scattering (DLS) and size exclusion chromatography (SEC), in comparison to commercial human IgG (Sigma) and IgM (Invitrogen). Samples were prepared by diluting various concentrations of TB-PCF in 70 μL of PBS, which were then loaded into disposable UV micro cuvettes (BRAND) for analysis using a Zetasizer Nano-ZS instrument (Malvern), following the manufacturer’s instructions. To estimate the size and purity, TB-PCF was loaded onto a Superose™ 6 Increase 10/300 GL column (Cytiva, USA) equilibrated with PBS pH 7.4 and connected to an ÄKTA pure (GE Healthcare, USA) FPLC system. The approximate size of TB-PCF fractionated by SEC was calculated by running protein standards (Sigma-Aldrich, 29,000-700,000 Da).

### GM1 ganglioside and sera binding assays

To assess the binding activity of TB-PCF to GM1 (monosialoganglioside GM1, Sigma), ELISA was performed as previously described [11]. Briefly, plates were coated with 5 μg/mL GM1 in PBS buffer (pH 7.4) and then incubated with TB-PCF at an initial concentration of 100 nM, followed by a 2-fold serial dilution. Blocking and detection (anti-mouse IgG-peroxidase) steps were in 5% non-fat dry milk protein solution in PBS. To visualise the peroxidase reaction, TMB substrate solution (Bethyl Laboratories, Inc) was added, and the reaction was stopped with 25 μL/well of 2 M H_2_SO_4_ prior to measuring absorbance at 450 nm using a plate reader (Tecan, UK). For TB patient sera reactivity with TB-PCF antigens, an ELISA was performed with immobilised TB-PCF, followed by addition of serial dilutions of sera from a TB patient or BCG vaccinated control (from our previous study (17)), and reactivity detected by anti-human IgG-peroxidase antibodies (Sigma).

### Binding and uptake of TB-PCF assay by confocal microscopy

To evaluate the binding and uptake of the TB-PCF construct by murine J774 macrophage cells, cells were incubated with either TB-PCF or a monoclonal murine IgG antibody (TBG65) (18) at 5µg/ml. The 100 µL mixture (50 µL of protein solution in PBS and 50 µL of cells in media) was incubated at 37 °C in a humidified 5% CO2 incubator for 4 h. Following the incubation with proteins, cells were fixed by washing with PBS and then incubated with 4% paraformaldehyde in PBS for 15 min at room temperature. After fixation, cells were permeabilised with 0.1% Triton X-100 in PBS for 10 min to allow antibody access to intracellular compartments. The cells were then washed with PBS and were incubated with an anti-mouse IgG FITC-conjugated antibody (BioLegend) at 1:250 dilution in PBS for 1 h at room temperature in the dark. After staining, the cells were washed thoroughly with PBS to remove excess antibody. Nuclei were counterstained with DAPI (ThermoFisher Scientific), and the samples were imaged using a Nikon A1R confocal microscope.

### Immunisation of C57BL/6 mice

C57BL/6 mice, aged 6–8 weeks, were sourced from Charles River (UK) and bred in-house at the University of Leicester Preclinical Research Facility. The mice (9–10 per group), comprising a mix of males and females, were divided into three groups (PBS, BCG, and TB-PCF). Three animals from each group (1 male, 2 females) were allocated for immunogenicity analysis, with the remaining mice (3 males, 3 females) used for Mtb challenge experiments. Sample size (n=6 per group for challenge experiments) was determined using a power calculation based on expected log_10_ CFU reduction with BCG as reported in prior studies (19). All animal procedures were conducted with approval from the University of Leicester Ethics Committee under a UK Home Office project license (Establishment License X1798C4D2) and followed the Animals (Scientific Procedures) Act 1986. Mice were immunised subcutaneously in the flank with 10 µg of TB-PCF or 5×10^5^ CFU of BCG Pasteur, in 0.1 mL. The control group received 0.1 ml PBS. The TB-PCF group then received two intranasal boosts using the same dose (10 µg, but in 0.05 ml) at 3-weeks intervals. Three weeks after the last immunisation, three mice from each group were euthanised by an intraperitoneal overdose of pentobarbital, and death confirmed by severing the femoral artery. Their tissues (sera, bronchoalveolar lavage fluid (BALF) and spleens) were used for immunogenicity analyses. The remaining mice (n=6) were subsequently used in an Mtb challenge experiment.

### Tissue collection and processing

Spleens, serum, lungs and BALF were collected from immunised mice from the immunogenicity arm of the *in vivo* experiment. Spleens were placed in 1 mL of MACS Tissue Preservation Media (Miltenyi Biotec), followed by rinse in PBS. The spleens were then mechanically disrupted by passing through a 40 µm cell strainer (Corning^®^) prewashed with 2 mL of cold R10 medium, consisting of RPMI with 10% FBS, 5 mM L-glutamine, 100 units/mL penicillin, 100 µg/mL streptomycin, 10 mM HEPES, and 50 µM 2-β-mercaptoethanol (Sigma). The resulting cell suspensions were further washed with 18 mL of R10 medium. Cells were then centrifuged at 250 rcf for 5 min at room temperature. To remove red blood cells, the cell pellets were treated with ammonium-chloride-potassium (ACK) lysis buffer (Sigma-Aldrich) for 5 min, followed by neutralisation with R10 medium. The isolated splenocytes were maintained in complete RPMI medium in a humidified incubator at 37 °C with 5% CO2 until use in antigen recall assays. Lungs were collected, minced into 1mm sections and digested in PBS containing 0.15 mg/mL DNase and 1 mg/mL collagenase on a rotating shaker at 37 °C for 45 minutes. The digested lung tissue was then passed through a 40 µm cell strainer followed by washing and red blood cell lysis as described earlier. Bronchoalveolar lavage fluid was obtained from the lungs of sacrificed mice by injecting 1 mL of sterile PBS into the lungs via a tracheal incision, followed by three rounds of flushing. The collected lavage was centrifuged at 1000 rcf, and the supernatant was stored at -20 °C until further use. Blood samples for serological analysis were collected via cardiac puncture. Blood was allowed to clot at room temperature for 1 h, followed by centrifugation at 1000 rcf for 10 min at 4 °C. The serum fraction was then recovered and stored at -20 °C until further analysis.

### *Ex vivo* vaccine efficacy by modified MGIA

The efficacy of cellular responses to TB-PCF vaccination in controlling mycobacterial infection *ex vivo* was determined by the Modified MGIA as described previously in Vergara et al, 2024 (20). Briefly, C57BL6/J bone marrow-derived macrophage cells (line BL/6-M) obtained from Kerafast^®^ (ENH166-FP) were maintained in DMEM (Sigma) supplemented with 10% FBS (Sigma) and 5 mM L-glutamine and seeded onto 48-well plates at a density of 500,000 cells per well. After 24 h incubation, cells were infected with H37Rv-Luciferase at a multiplicity of infection (MOI) of 2:1. Infected cells were then harvested by aspirating culture media followed by enzyme-free dissociation buffer (ThermoFisher Scientific). H37RV-Luciferase-infected BL/6-M cells were then co-cultured with splenocytes single cell suspensions from immunised experimental animals for 5 days at 37 °C 5% CO2 in a humidified incubator. Extracellular H37RV-Luciferase were killed by incubating samples with 50 µg/ml amikacin for 2 h, followed by washing with PBS and cell lysis with 200µl DH2O + 0.1% Triton-X100 per sample. Luciferase substrate working solution was prepared by making a 1:10 dilution of a decanal stock solution (1% decanal in ethanol) in PBS. Cell lysates were added to 1 ml of luciferase working solution and immediately read on a Junior LB 9509 Portable Luminometer (Berthold Technologies GmbH & Co.) set to 30 second exposure time. Data were expressed as relative luminescence units (RLU).

### Aerosol Mtb challenge and CFU enumeration

The Mtb strain H37Rv was cultured in Proskauer Beck medium with 0.05% Tween-80 until mid-log phase, then frozen in 1 mL aliquots at -70 °C. In a containment level 3 facility, mice were challenged with approximately 100 colony-forming units (CFU) using a custom aerosol chamber (Walker Safety Cabinets Ltd., Glossop, UK) equipped with a “jet in air” venturi nebuliser. On day 23 post-challenge, mice were euthanised via an intraperitoneal overdose of pentobarbital, and their lungs were aseptically harvested to assess mycobacterial burden. For bacterial enumeration, lung homogenates were prepared by GentleMACS Dissociator (Myltenyi Biotec) in solution containing 0.1% Triton X-100. Homogenates were plated in technical duplicates on Middlebrooks 7H11 agar (BD Biosciences) supplemented with oleic acid-albumin-dextrose-catalase (OADC) (Millipore), glycerol and Selectab (Mast Diagnostics). CFUs were counted after 4 weeks incubation at 37 °C.

### Antibody quantification in sera and BALF

An ELISA was performed to measure ESAT6-CFP10 antigen-specific IgG and IgA titres in the serum and BALF of vaccinated mice. 96-well microtiter plates (Nunc) were coated with 5 µg/mL of ESAT6-CFP10 in coating buffer (0.1M NaHCO_3_, pH 9.6) and incubated at 37 °C for 2 h. After removing the coating solution, the plates were washed three times with distilled water. The wells were then blocked with blocking buffer (5% skimmed milk powder in PBS) for 2 h at 37 °C. Following another set of three washes with distilled water, serial two-fold dilutions of mouse serum (starting from 1:100) and BALF (starting from 1:5) samples were added to the plates and incubated overnight at 4 °C. The next day, plates were washed three times before adding respective secondary antibodies (peroxidase-conjugated anti-mouse IgG1, IgG2a, IgG2b and IgA, Sigma and The Binding Site) at 1:1000 dilution in blocking buffer and incubated for 2 h at room temperature. After five washes, the plates were developed with TMB substrate solution (Bethyl Laboratories, Inc), and the reaction was stopped by addition of 25 μL/well of 2 M H_2_SO_4_ prior to measuring absorbance at 450 nm using a plate reader (Tecan, UK).

### T_RM_ analysis and T cell antigen recall assay

For detection of CD4+ lung tissue resident memory (T_RM_), T cells, 4 million mononuclear lung cells were plated per well in 96-well U bottom plates for surface staining with CD44-FITC, CD4-PerCP/Cy5.5, CD3-APC, CD62L-PE, CD69-PE-Cy7, CD103-BV421 (BioLegend, San Diego, CA, USA) and Fixable Viability dye BV510 (BD Biosciences). T cell antigen recall assays were performed using single-cell suspensions from splenocytes seeded into 96-well flat-bottom tissue culture plates at a density of 1 × 10^6^ cells/well in R10 media and stimulated with 5 µg/mL ESAT6-CFP10 or medium control. After 5 days of culture, cells were harvested and transferred to 96-well U-bottom plates for subsequent staining. The cells were washed with phosphate-buffered saline (PBS) and then incubated with Fc Block (Human TruStainFcX™ anti-mouse CD16/32) for 2 h at 4 °C, alongside fluorochrome-conjugated secondary antibodies specific to CD3-APC/Cy7, CD4-PerCP-Cy5.5, CD8-AF700, CD44-FITC, and CD62L-PE (Biolegend), as well as fixable viability dye-BV510. Following two PBS washes, cells were fixed and permeabilised using a Fix/Perm kit according to the manufacturer’s instructions (ThermoFisher Scientific). The expression levels of intracellular cytokines were analysed by flow cytometry after incubation with specific anti-mouse antibodies at 4 °C for 1 h. For Panel A, secondary antibodies targeting TNF-α-APC, IFN-γ-PE-Dazzle 594, and IL-2-BV605 were used, while for Panel B, secondary antibodies targeting IL-17-PE-Cy7, IL-2-BV605, and Ki67-APC were employed. All antibodies were diluted in permeabilisation buffer included in the Fix/Perm kit. All flow cytometry data was acquired on a CytoFLEX cytometer (Beckman Coulter) and analysed using FlowJo V10 (FlowJo LLC). Fluorescence minus one (FMO) control was used to set the gates and determine positive populations for each marker. For antigen recall analysis, T effector memory (T_EM_) cells were identified by gating on CD3, followed by CD4 and CD8, and finally CD44^hi^ and CD62L^lo^ expression.

### Cytokine quantification in culture supernatants

Cell supernatants from antigen recall assays were stored at -80 °C and later analysed using ELISA kits (ThermoFisher Scientific) to quantify IFN-ɣ, TNF-α, IL-4, and IL-10 according to the manufacturer’s instructions. Samples were diluted 1:20 for IFN-ɣ and 1:5 for TNF-α, IL-4, and IL-10. Cytokine concentrations were determined by interpolating TMB substrate absorbance readings at 450 nm from a standard curve, then multiplying by the respective dilution factor.

### Aerosolisation of TB-PCF

TB-PCF was aerosolised using the commercially available Omron Micro Air U22 electronic mesh nebuliser. 1 ml of 100 µg/ml TB-PCF in PBS pH 7.4 or PBS containing 0.05% Tween-80 was loaded into the fluid chamber with the exhaust sealed onto a pre-weighed glass collection vessel at a 45° angle. Nebulisation was turned on until the chamber emptied, followed by the collection vessel being sealed and placed in ice to condense the sample. Protein recovery was calculated by measuring the volume of recovered sample and protein concentration by OD280.

### Mucosal cytotoxicity assay

To establish the alveolar epithelial model, human alveolar epithelial cells (hAECs) obtained from Generon, UK, were first expanded in Pneumacult media (Stemcell Biotechnologies) to reach 80% confluency. The cells were then detached using Trypsin-EDTA (Merck) and washed with PBS before being resuspended in Endothelial Growth Media 2 (EGM2) (Promocell) containing Primocin (Invivogen). Approximately 100,000 cells were seeded onto the apical surface of 1µm pore size PET tissue culture inserts (33 mm^2^) within 24-well tissue culture plates. The seeding volume was maintained at 100µl on the apical side and 650µl EGM2 was added to the basolateral compartment. Over the course of seven days, cells were allowed to form a monolayer with media refeeding performed at day four for both compartments. At day seven, the apical supernatant was removed to create an air-liquid interface (ALI) and promote differentiation of the hAECs into a functional epithelial layer.

Membrane integrity was measured by Transepithelial Electrical Resistance (TEER) using an Millicell ERS-2 Voltohmmeter (Millipore) after submerging the apical side in 200µl PBS and equalising alveolar plates to room temperature, with results expressed as Ω/cm^2^, and readiness of hAEC monolayers for experimentation defined as the point at which TEER measurements plateau. For assessment of interaction of samples to the hAEC monolayer, PBS, IgG, CTB, ESAT6-CFP10 or TB-PCF (10µg/ml) were added to the apical side in 100µl PBS and incubated for 48 h. Apical and basolateral supernatant was collected and stored at -80 °C, while TEER of monolayers was assessed to determine effects of treatment on membrane integrity. From apical and basolateral supernatant, cell viability was determined by Lactate Dehydrogenase (LDH) release using the chromogenic CyQUANT LDH assay (Invitrogen).

### Statistics

Statistical analyses were performed using GraphPad Prism V10. One-way ANOVA multi-comparison test followed by Tukey’s *post hoc* correction test were performed to determine statistically significant differences between various conditions, as defined by p ≤ 0.05. Confidence levels are indicated by single or multiple asterisks, as indicated in figures. Bars represent means of biological or technical repeats and error bars represent standard error or deviation of the mean, as indicated. Specific circumstances are described in detail in the results and discussion sections.

## Results

### Construction and structural modelling of TB-PCF

**Figure 1:**
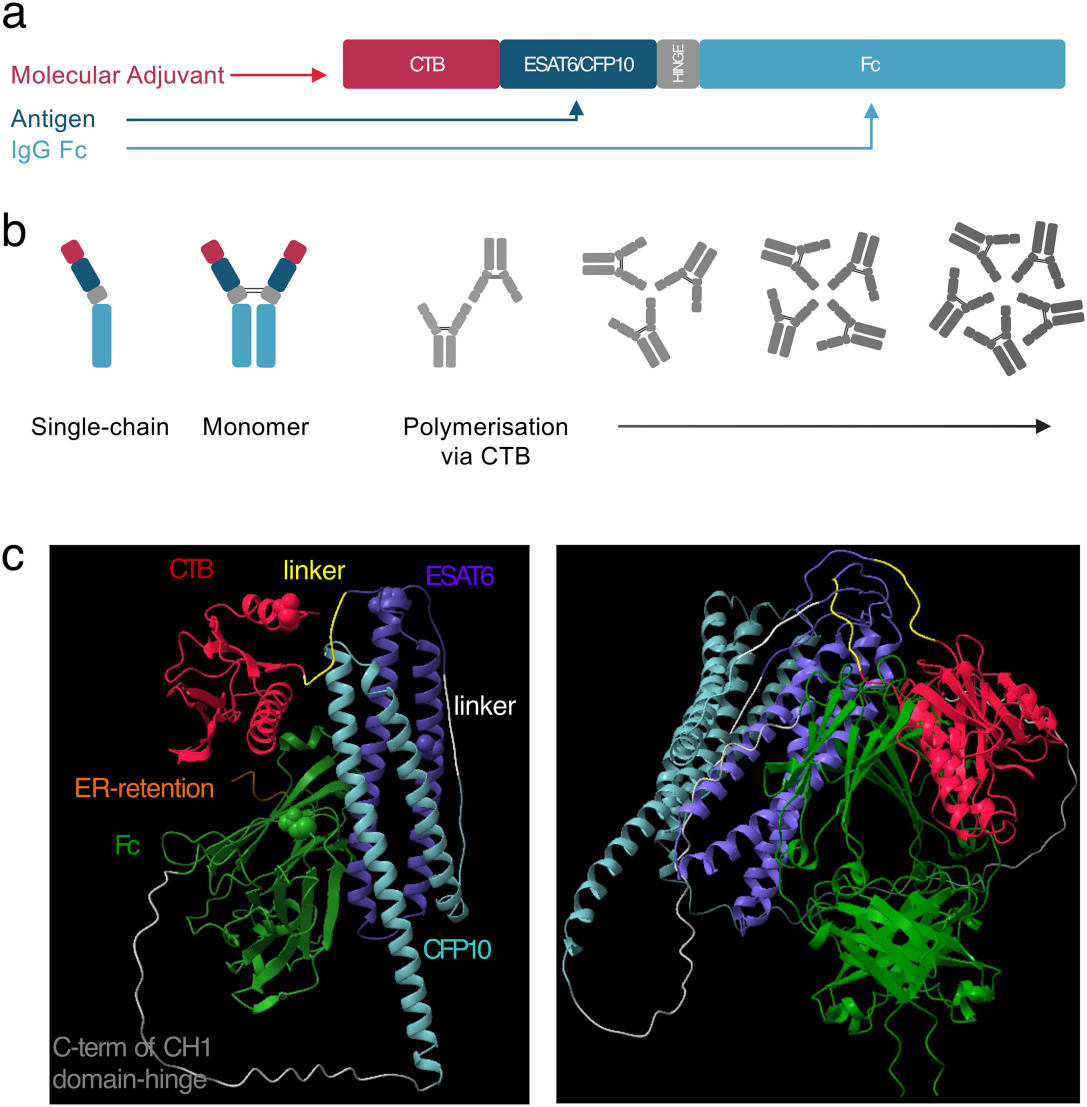
Structure and assembly of TB-PCF. **(a)** Building blocks of TB-PCF, showing the three components sequentially: CTB, mouse IgG2a-Fc (Fc) and ESAT6-CFP10 fusion protein; **(b)** The diagram shows a monomer that subsequently forms a homo-dimer via a disulfide bond between the two Fcs, and then polymerisation via a non-covalent bond of CTB to form a pentameric structure; shown are also various intermediate forms **(c)** AlphaFold2 and ChimeraX modelling of TB-PCF monomer (left) and dimer (right) showing structural display of various domains and the linkers. The C-terminal ER-retention peptide (SEKDEL) is shown in orange. Four arrows indicate a putative N-glycosylation site in each domain. The homodimer (right) shows two copies of all components paired by a disulfide bond between Fc-Fc. Colours: red, CTB; green, IgG-Fc; light blue, CFP10; purple, ESAT6.

The TB-PCF polypeptide incorporates three key components: non-toxic cholera toxin B subunit (CTB) as molecular adjuvant, the antigen of choice (in this case ESAT6-CFP10 fusion protein of Mtb) and the IgG-Fc fragment (derived from mouse IgG2a) as the APC targeting moiety, for enhanced endocytosis through IgG Fc receptors (**Figure 1a**). Short linkers are also incorporated between CTB and antigen, between ESAT6 and CFP10 and between antigen and Fc, for added structural flexibility. SEKDEL peptide is included at the C-terminus of the molecule, for enhanced endoplasmatic reticulum (ER) retention. The protein is expressed as a single polypeptide (monomer) which then dimerises through Fc pairing via disulfide bonds, much like antibodies do (**Figure 1b**). Further multimerisation is achieved through CTB aggregation to theoretically form CTB pentamers [14], with each pentamer incorporating ten copies of antigen. Our AlphaFold2 modelling of tertiary structure of TB-PCF predicts surface display of the antigen and full accessibility of CTB and Fc domains in either monomeric form (**Figure 1c** left) or when dimerised through Fc (**Figure 1c** right), for interaction with their target receptors, gangliosides and IgG Fc receptors, respectively. The confidence level of prediction is overall high, as expressed by pLDDT and PAE scores (**Supplementary Figure 1**). pLDDT (predicted Local Distance Difference Test) is a per-residue score indicating local confidence, showing that the model accurately predicts the position of each amino acid relative to its neighbours in both Fc and CTB, and to a large degree also for the ESAT6-CFP10 (**Supplementary Figure 1** left). PAE (Predicted Aligned Error) is a matrix showing global confidence, representing the expected error (in Ångströms) between pairs of residues if the predicted structure were aligned to the true one for each component, revealing reliable domain arrangements for CTB, Fc and ESAT6-CFP10 (**Supplementary Figure 1** right). Furthermore, when predicted ESAT6 and CFP10 structures within TB-PCF were compared to published (21) X-ray 3D structure of the heterodimer, a near identical agreement was observed for CFP10 and a good agreement for ESAT6 (**Supplementary Figure 2**). Together, these results confirm the high accuracy and structural reliability of the modelled TB-PCF structure, indicating that the antigen is likely correctly structured within the vaccine construct and therefore available to immune cells in a biologically relevant and accessible form.

### Biophysical properties of TB-PCF

**Figure 2:**
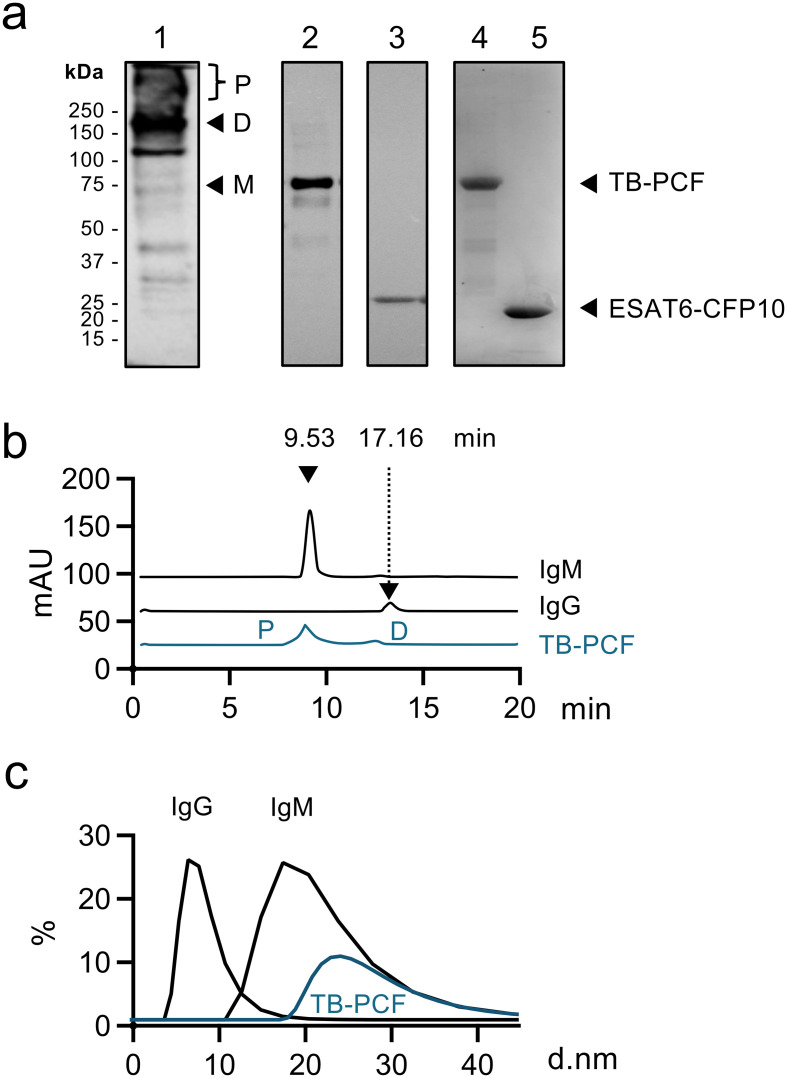
Characterisation of TB-PCF by western blot and coomassie staining **(a)**, SEC **(b)** and DLS **(c)**. **(a)** Western blot analysis to detect 1 μg of TB-PCF under nonreducing conditions with anti-ESAT6 antibodies (lane 1). The expected computational size of 61 kDa for the monomeric TB-PCF (without 4 N-glycans). M, D, P represent monomer, dimer and polymer, respectively. Under reducing conditions (lane 2), only monomers are detected, while ESAT6 specificity of the antibody is confirmed with ESAT6-CFP10 fusion protein (lane 3). **(b)** Size analysis by size-exclusion chromatography (SEC). TB-PCF, human IgM and IgG were compared and elution profiles overlayed. Profile for TB-PCF shows two main peaks at 9.5- and 13-min elution time, representing 63.72% and 29.83% of the loaded protein, respectively. Based on similarity to IgM and IgG elution times, these likely represent polymers (P) and dimers (D). **(c)** Particle size analysis of TB-PCF using dynamic light scattering (DLS). TB-PCF, IgM (900 kDa) and IgG (150 kDa) were directly compared.

The expected computational size of monomer TB-PCF by Protparam at Expasy is predicted at 61.3 kDa, not accounting for the four potential N-glycosylation sites (one in CTB and Fc each, and two in ESAT6-CFP10, respectively). The purified TB-PCF was analysed by SDS-PAGE and Western blot under both non-reducing (NR) and reducing (R) conditions (**Figure 2a**), using antibodies specific for ESAT6 antigen. Under non-reducing conditions, presence of 150 kDa molecular form (likely dimer) and higher molecular weight bands were observed (**Figure 2a**, lane 1), indicative of the presence of polymeric TB-PCF forms. However, the denaturing conditions of the gel may have affected the integrity of these complexes, resulting in apparent molecular weights that are smaller than those in the native state. Under reducing conditions, a band at 75 kDa was detected (**Figure 2a**, lane 2), corresponding to the expected monomeric form of TB-PCF, including likely glycosylation. To confirm the specificity of the anti-ESAT6 antibody, ESAT6-CFP10 was analysed under similar conditions, showing a band at 25 kDa, consistent with the expected size of the fusion protein (lane 3). Coomassie staining of purified proteins, also revealed the correct size protein bands for monomeric TB-PCF under reducing conditions (lane 4) and ESAT6-CFP10 fusion protein (lane 5). It is worth noting, that in the context of TB-PCF, the monomer refers to the single chain polypeptide consisting of one of each CTB, Ig-Fc and antigen molecules, and is different from antibody monomer, where two Ig-Fc molecules are linked together by disulfide bonds.

Size exclusion chromatography (SEC) of TB-PCF under native conditions was performed alongside monomeric human IgG and pentameric IgM, as controls (**Figure 2b**). The elution profile revealed a peak at ~9.5 min for TB-PCF, which coincided with the elution time of pentameric IgM, strongly suggesting that TB-PCF forms polymers. In contrast, the monomeric IgG control eluted at ~13 min, consistent with its smaller size. Dynamic light scattering (DLS) further corroborated these findings, as the particle size of TB-PCF was more comparable to pentameric IgM than to monomeric IgG (**Figure 2c**), providing additional evidence for the formation of polymeric complexes by TB-PCF under native conditions.

### Functional *in vitro* characterisation of TB-PCF

**Figure 3:**
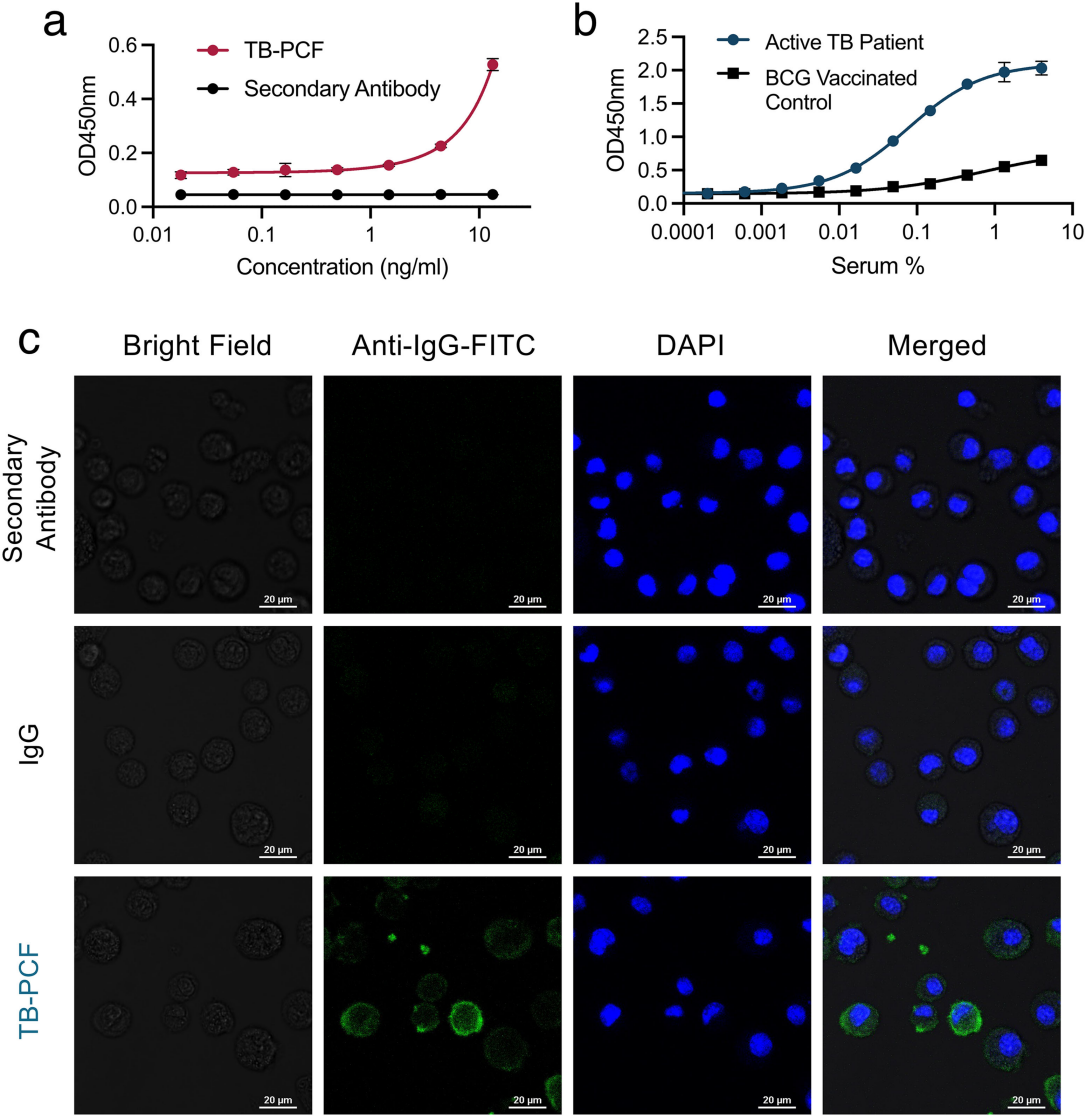
Functional characterisation of TB-PCF **(a)** Binding of increasing concentrations of TB-PCF to immobilised GM1 ganglioside (via CTB),compared to secondary antibody alone; **(b)** Human serum from a TB patient and BCG vaccinated control reactivity with immobilised TB-PCF, to confirm accessibility of antigens to antibodies and B cells; shown are increasing concentrations (expressed as % vol/vol dilution) of sera. ESAT6-CFP10 are not present in BCG and detected low reactivity is likely non-specific background caused by high serum concentration. **(c)** Binding to and uptake of TB-PCF by J774 macrophage cells by confocal microscopy. DAPI stain and anti-IgG-FITC were used to stain the nucleus (blue) and internalised protein (green); monomeric mouse IgG was used as the comparator and the secondary antibody alone was used as the negative staining control, respectively. Panels (from left to right) show bright field, anti-IgG-FITC and DAPI staining, while the last panel represents the merged image. Representative of several images taken.

*In vitro* functional characterisation of the purified TB-PCF was conducted to assess its binding properties and antigen availability. First, an ELISA was performed to evaluate the binding of TB-PCF to GM1, the receptor for the CTB protein on TB-PCF (**Figure 3a**). This assay confirmed the correct expression and availability of the CTB protein in the native form of TB-PCF, demonstrating a concentration-dependent binding to immobilised GM1 protein. To further confirm the correct expression and availability of the ESAT6-CFP10 antigens within TB-PCF, serum antibodies from a TB patient were tested for ability to bind TB-PCF. The results showed a concentration-dependent binding of serum antibodies to TB-PCF (**Figure 3b**), indicating that the antigens are accessible and can be recognised by antibodies from TB patients. In contrast, serum from BCG-vaccinated, TB-negative hosts exhibited only background binding to TB-PCF, and only at high serum concentration, i.e. 1-10% vol/vol.

To investigate the binding and uptake of TB-PCF by phagocytic cells, J774 murine macrophages were incubated with either monomeric IgG or TB-PCF. Following incubation, the cells were permeabilised and stained with anti-IgG FITC, while DAPI and phalloidin stains were used to visualise the nucleus and cell structure, respectively. The results of confocal analysis demonstrated significantly greater binding and uptake of TB-PCF compared to monomeric IgG, indicating that the polymeric nature of TB-PCF enhances its phagocytosis efficiency (**Figure 3c**). This suggests that TB-PCF’s polymeric structure may play an important role in its interaction with antigen-presenting cells via the Fc receptors, potentiating antigen presentation.

### Humoral responses to TB-PCF and lung memory T cells in vaccinated mice

**Figure 4:**
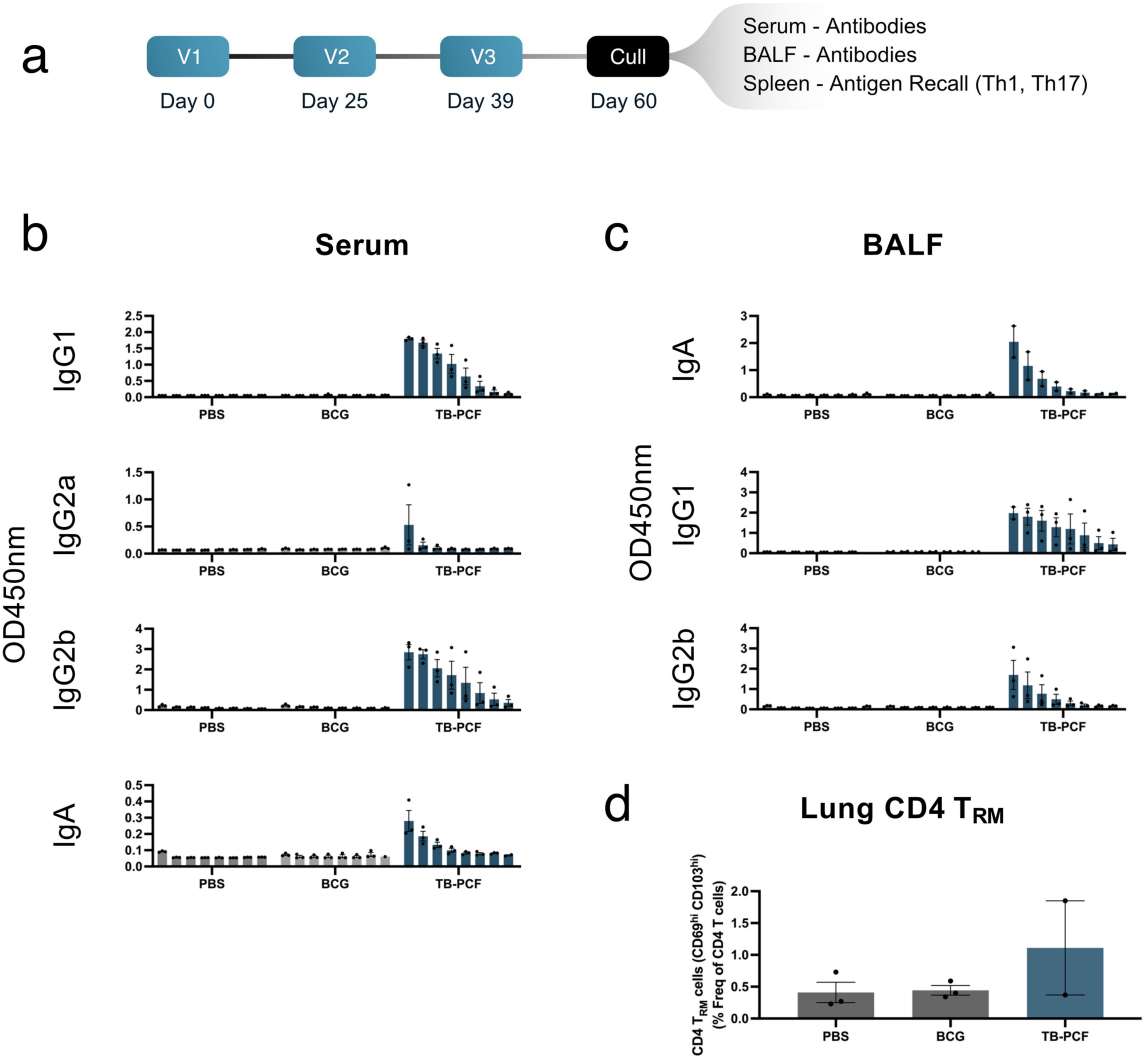
Analysis of serum and BALF antibody responses in vaccinated mice and lung T_RM_ responses. **(a)** schematic depiction of immmunisation regimen and immunological readouts. **(b)** serum responses to ESAT6-CFP10 fusion protein measured by ELISA; shown are responses for IgG subtypes and IgA. **(c)** BALF antibody responses for IgA, IgG1 and IgG2b. Shown are 3-fold serial dilutions starting from 1:100 (serum) or 1:5 (BALF). **(d)** total levels of CD4+ TRM were assessed by CD44+ CD62L- and CD69+ CD103+ cells as a proportion of total CD4 T cells in the lung tissue. Mean presented and error bars represent SEM from biological triplicate readouts.

Mice were immunised with either PBS, BCG or TB-PCF, and their antibody responses analysed in sera and BALF, by ELISA (**Figure 4**). Fusion protein ESAT6-CFP10 was immobilised on the plate and humoral responses detected using anti-mouse secondary antibodies. In serum, antigen-specific humoral responses were observed for TB-PCF vaccinated animals only, for all IgG subtypes tested, as well as for IgA. Notably, IgA and IgG responses were also observed in the BALF of TB-PCF vaccinated mice, but not in BCG group. This was expected, as ESAT6 and CFP10 antigens are not present in BCG, due to the absence of RD1 in the vaccine strain (22). Lung resident memory T (Trm) cell analysis in vaccinated mice revealed no detectable CD8+ Trm in any of the groups, and only a modest, statistically nonsignificant increase in CD4+ Trm in TB-PCF group. Thus, the proportion of CD4+ lung T_RM_ cells (CD69+ CD103+) in the lung was numerically higher in the TB-PCF group compared to PBS and BCG controls (**Figure 4d**), suggesting a potential trend toward enhanced local T-cell retention.

### Cellular responses to TB-PCF vaccination in mice

**Figure 5:**
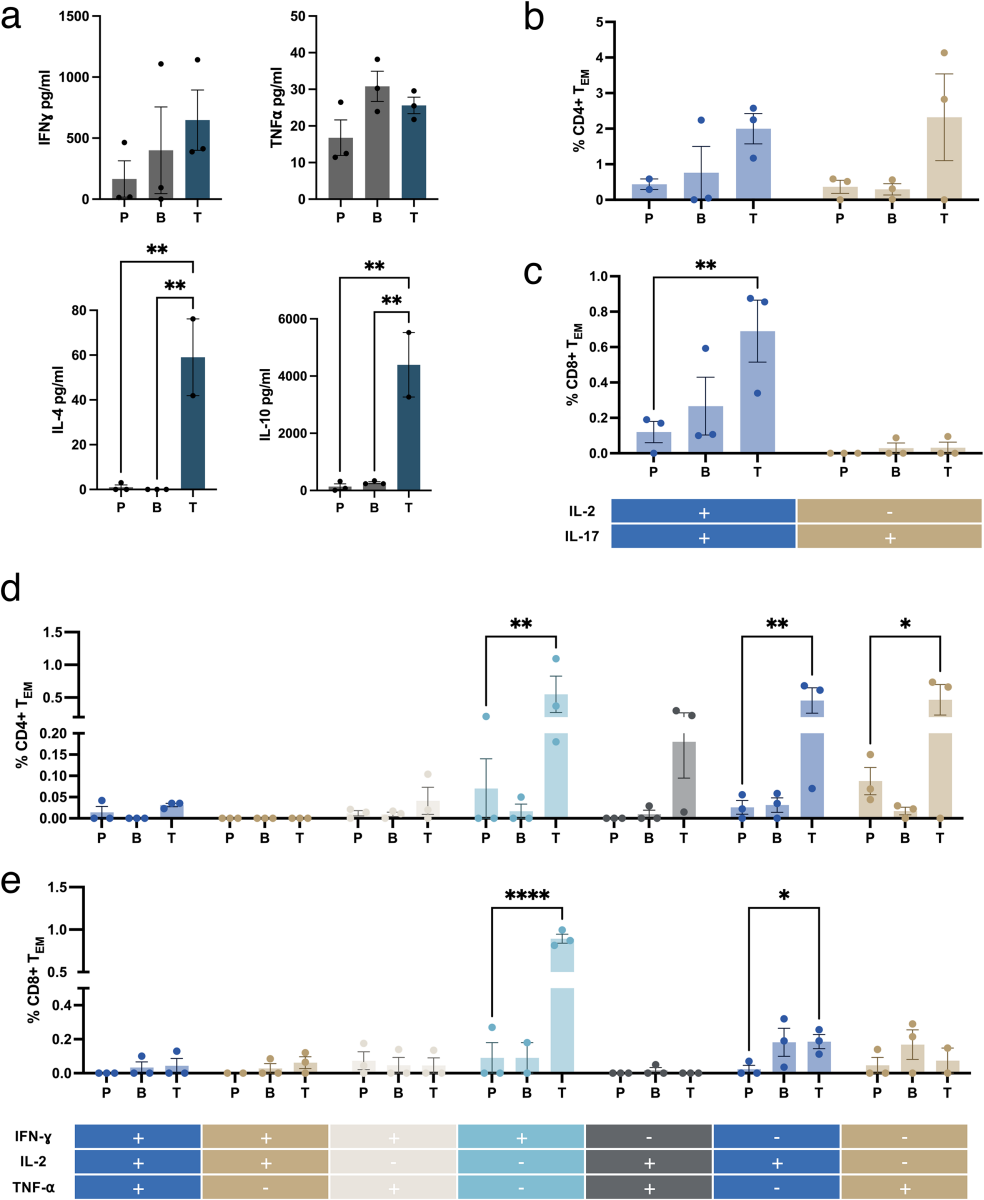
Antigen recall cellular responses from splenocytes of vaccinated mice. **(a)** IFN-γ and TNF-α responses in supernatants from 5-days ESAT6-CFP10 stimulated splenocyte cultures (by ELISA). **(b, c)** Percentages of CD4+ **(b)** and CD8+ **(c)** responding effector memory T cells (T_EM_) producing IL-2, IL-17 or both, after antigen restimulation. d and e) Percentages of CD4+ **(d)** and CD8+ **(e)** T_EM_ expressing single or multiple cytokines (IFN-γ, TNF-α and IL-2) by intracellular staining. Shown are means + SEM from three biological replicates and P, B and T denote PBS, BCG or TB-PCF immunised mice, respectively. Tables under b and c, and d and e, denote cytokine positivity (+) or negativity (–). Statistical analysis conducted by One-way ANOVA and Tukey’s *post-hoc* test, with *denoting p<0.05, **p<0.01 and ****p<0.0001.

To evaluate cellular immune responses elicited by TB-PCF vaccination, an antigen recall assay was conducted using splenocytes from vaccinated animals. These cells were stimulated with the ESAT6-CFP10 antigens, and the resulting supernatants were analysed for cytokine levels by ELISA. Notably, TB-PCF-vaccinated animals exhibited elevated levels of IFN-ɣ (**Figure 5a**), a cytokine associated with a protective host phenotype against Mtb infection (23). Increased levels of IL-4 and IL-10 were also observed in samples from TB-PCF-vaccinated animals, indicating a regulated Th1/Th2 response, while no difference in TNF-α levels were observed compared to controls.

To further characterise the immune response, intracellular cytokine staining was employed to assess Th17 and Th1 responses in CD4+ and CD8+ T cells. TB-PCF vaccination induced Th17 responses, as evidenced by the presence of IL-2 and IL-17-positive cells in both CD4+ and CD8+ compartments (**Figures 5b, c**). Additionally, TB-PCF vaccination significantly increased the proportion of IFN-ɣ-positive cells in both CD4+ (**Figure 5d**) and CD8+ (**Figure 5e**) compartments, and IL-2 and TNF-α single positive cells in CD4+ compartment only. Th1 responses were further characterised by increased secretion of IL-2 and TNF-α double positive cells in the CD4+ compartment, indicating polyfunctionality.

### Protective potential of TB-PCF induced immunity in mice

**Figure 6:**
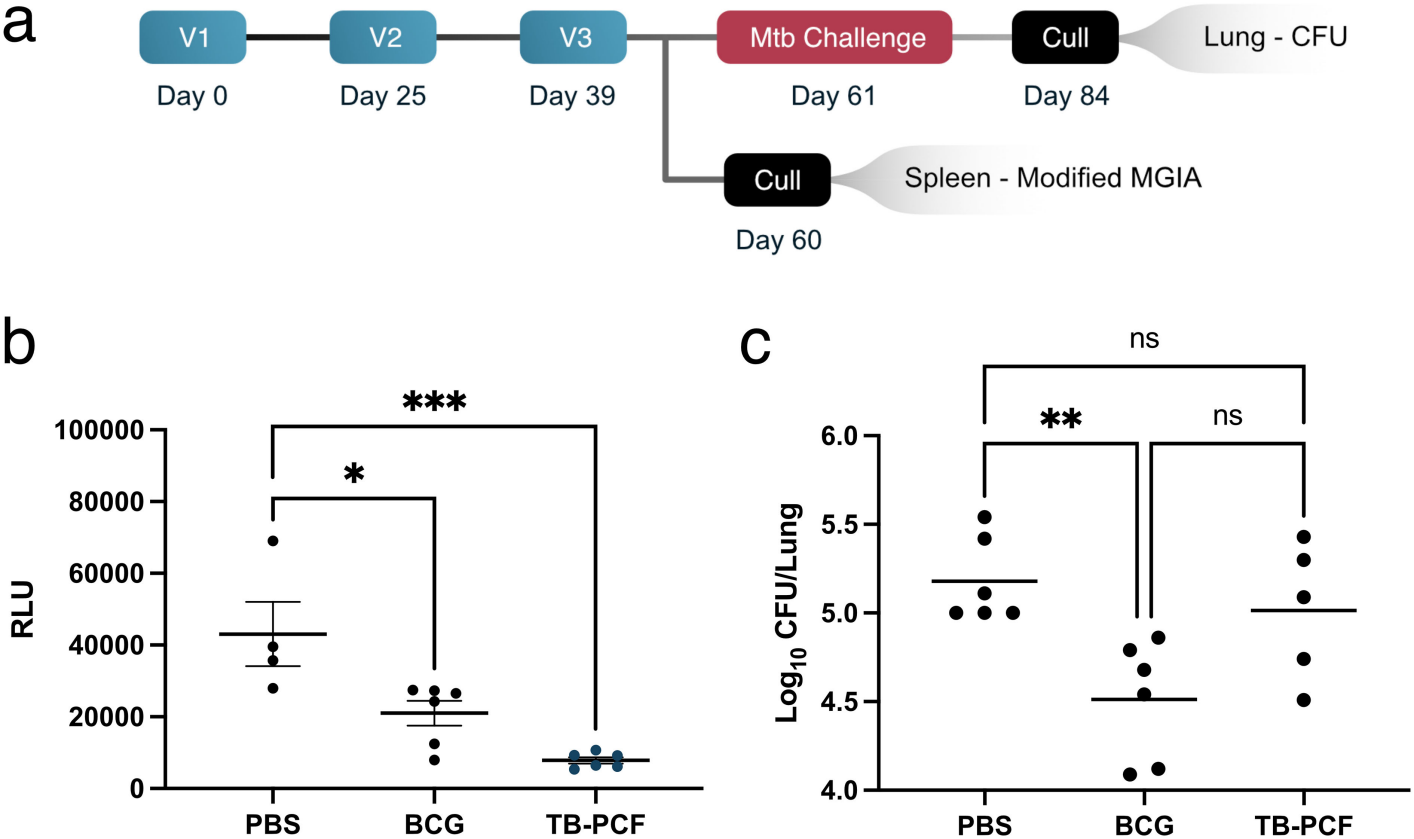
Assessment of protective efficacy of TB-PCF. **(a)** The experimental plan showing immunisation regimen and two different efficacy readouts. **(b)** Assessment of vaccine efficacy by modified-MGIA in splenocyte cultures supplemented with mouse BL/6-M mouse macrophage cells and infected with H37Rv-luciferase strain of Mtb (n=3), readouts in relative luminescence units, RLU). **(c)** Log_10_ lung CFU of immunised mice (n=6) after aerosol infection (23 days). Shown are individual mouse readings and the horizontal line represents the mean. Statistical analysis conducted by One-way ANOVA and Tukey’s *post-hoc* test, with *denoting p<0.05, **p<0.01 and ***p<0.0005, ns, nonsignificant.

To test the protective potential of TB-PCF vaccine construct in mice, two approaches were undertaken (**Figure 6a**). In the first, we performed the modified MGIA assay (20) with immune splenocytes infected with H37Rv Mtb recombinant strain expressing luciferase enzyme. This assay enables to quantify the capacity of splenocytes to kill infected target cells and is a proxy for vaccine efficacy *in vivo*. Splenocytes from both BCG and TB-PCF immunised mice exhibited statistically significant bacterial growth inhibition compared to mock-immunised mice, with TB-PCF showing a trend towards greater inhibition (**Figure 6b**). In the second approach, immunised mice were challenged with aerosolised Mtb and their lungs analysed for bacterial burden by the standard CFU plating assay. As shown in **Figure 6c**, only BCG induced statistically significant reduction of lung bacterial burden, while TB-PCF only showed a nonsignificant trend towards reduction. Therefore, the CFU assay did not replicate the outcome of the MGIA assay. It is worth noting that the apparent intragroup variability may have contributed to these disparate outcomes between the two assays, potentially amplified by the modest sample size of n=6 per group for the challenge study. However, considering that the *in vivo* protection is clearly the more relevant assay, more work is needed to fully establish the protective capacity of this vaccine approach, including incorporating additional antigens or combinations.

### Aerosolisation and alveolar epithelial safety profile of TB-PCF

**Figure 7:**
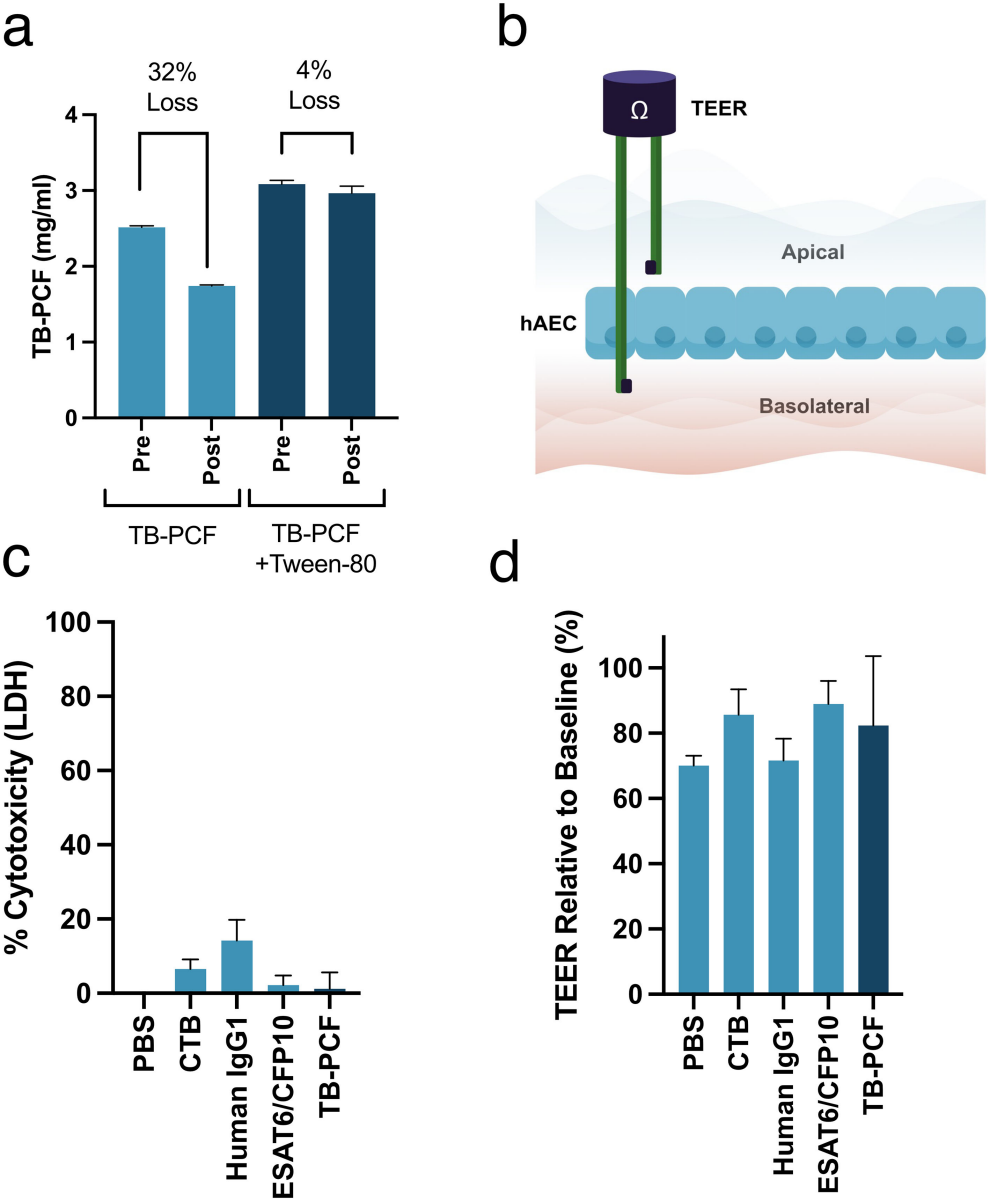
Feasibility of aerosolisation of TB-PCF and mucosal safety profile. **(a)** protein content before (‘pre’) and after (‘post’) aerosolisation in Omron Micro Air U22 electronic mesh nebuliser, with or without addition of 0.05% Tween-80 as excipient; percentages above bars indicate protein loss. **(b)** schematic depiction of human alveolar epithelial cells (hAEC) monolayer, with integrity measured by Transepithelial Electrical Resistance (TEER). **(c)** cytotoxicity of TB-PCF measured by lactate dehydrogenase (LDH) assay, at 24 h post treatment; PBS, CTB, human IgG and ESAT6-CFP10 were used as comparators and controls. **(d)** Monolayer integrity measured by TEER in the same cultures. Error bars represent SEM from three technical repeats.

Finally, to evaluate the feasibility of TB-PCF for mucosal delivery, we assessed its properties following aerosolisation using an Omron Micro Air U22 nebuliser. TB-PCF was aerosolised, and protein recovery was quantified by ELISA. Aerosolisation alone resulted in a 32% loss of protein from the condensate. However, the addition of Tween-80, a non-ionic detergent commonly used as an excipient in drug formulations, increased protein recovery to 96% (**Figure 7a**). Furthermore, considering that TB-PCF contains CTB as molecular adjuvant, we also evaluated its safety for aerosolised delivery. We utilised human alveolar epithelial cells grown in 1µm transwell inserts to form polarised tight monolayers under air-liquid interface conditions, to mimic the alveolar epithelium (**Figure 7b**). After 48 h of incubation with TB-PCF and various controls in the apical compartment, lactate dehydrogenase (LDH) activity and transepithelial electrical resistance (TEER) were measured in the apical and basolateral supernatants. The LDH levels, indicative of cytotoxicity, were comparable between TB-PCF-treated samples and controls, suggesting no significant cytotoxicity (**Figure 7c**). Similarly, TEER measurements, reflecting the integrity of the epithelial barrier, showed no significant difference from baseline readings for any treatment group compared to the PBS control (**Figure 7d**). These results indicate that TB-PCF does not compromise the integrity of the alveolar epithelium and is well-tolerated when administered mucosally, supporting its favourable safety profile as a mucosal vaccine delivery system.

## Discussion

In this study, we demonstrated that systemic priming and mucosal boosting with TB-PCF vaccine construct in mice elicited antigen-specific antibody responses in serum and bronchoalveolar lavage fluid, elevated levels of IFN-ɣ, robust Th17 and Th1 responses, with increased frequencies of various cytokine-positive CD4+ and CD8+ T cells. We observed a numerical (although not statistically significant) increase in lung CD4+ T_RM_ cells in TB-PCF-vaccinated mice compared to PBS and BCG-vaccinated controls. Given the supporting role of T_RM_ in early control of Mtb infection (24), this trend warrants further investigation with larger sample sizes to assess T_RM_ persistence and functional capacity. Splenocytes from TB-PCF-vaccinated mice showed significant bacterial killing in a modified mycobacterial growth inhibition assay, trending higher than BCG, but *in vivo* challenge test showed only BCG achieved statistically significant reduction in lung bacterial burden. It is important to note that the modified MGIA primarily reflects systemic T cell-mediated immunity and may not fully capture the functional capacity of tissue-resident or mucosal T cells which are key mediators of protection at the site of Mtb entry (24). While these outcomes show a promising potential of this new vaccination approach for TB, they also indicate the need for further improvements before potential translation towards clinical testing.

TB remains a pressing global health challenge, and while the BCG vaccine is safe and effective for infants against disseminated TB, its protection wanes over time and it fails to protect against pulmonary TB (25, 26). This may be in part at least due to its failure to induce robust mucosal immunity (27), being a systemically delivered vaccine. Systemic administration of the BCG vaccine, typically via the intradermal route, primarily stimulates central memory T cells that circulate in the blood and lymphatic system rather than residing in the mucosal tissues where Mtb first enters (28). A critical limitation of this route is its failure to induce robust populations of lung-resident memory T cells (TRM), which are essential for immediate, local recognition of the pathogen (27). Additionally, systemic vaccination does not effectively stimulate the production of mucosal IgA, the primary antibody responsible for neutralising pathogens at mucosal surfaces (29), and also shown to protect against TB when delivered passively (30, 31). Consequently, while intradermal BCG protects against disseminated disease, it remains suboptimal for preventing pulmonary TB because the protective immune effectors are not strategically positioned at the site of infection. While this can be overturned by altering route of BCG administration, such as giving it intravenously (32) or via respiratory route (33), it is clear that next-generation TB vaccines are required, that should ideally not only match BCG in many aspects of its performance but critically, offer enhanced mucosal immunity. Many candidates for mucosal TB vaccination have been proposed, but none has reached licensure yet (34).

Our newly developed PCF mucosal vaccine platform can aid in the development of next-generation TB vaccines, aiming to boost mucosal immunity. While the PCF vaccine platform has previously demonstrated efficacy against viral pathogens such as dengue (13) and SARS-CoV2 (14), its effectiveness against bacterial pathogens, including Mtb, has not been explored. In this study, we report the first evidence that this vaccination approach elicits systemic and mucosal immune responses and exhibits some protective effects against Mtb, though this may be largely dependent on the choice of antigen(s).

TB-PCF was generated by incorporating two virulence-associated antigens of Mtb, ESAT6 and CFP10 into the PCF vaccine platform, where the fusion protein is flanked by CTB (N-terminal) and IgG Fc (C-terminal). The single chain polypeptides expressed in *N. benthamiana* plants were then assembled into antibody-like dimeric structures, which further undergo noncovalent pentamerisation mediated by the CTB component *in planta*. This was confirmed experimentally by various *in vitro* characterisation assays including SDS-PAGE, SEC and DLS, showing a high abundance of polymeric structures in the purified TB-PCF preparations. We hypothesised that the polymeric structures of TB-PCF can improve binding and uptake of the molecule by APC via host Fc receptors, as is the case with immune complexes, and this was visualised by confocal microscopy in binding/uptake assays.

The adjuvanticity of TB-PCF arises from both IgG-Fc and CTB components, with the former allowing for interaction and subsequent uptake of TB-PCF via Fc receptors on APC, while the inclusion of CTB serves both to promote polymerisation, as well as provide local adjuvanticity through binding to gangliosides on epithelial cells (35). The most significant evidence for CTB safety is its long-standing use in the licensed oral cholera vaccine, Dukoral^®^, which contains 1 mg of recombinant CTB (36). The favourable pre-clinical safety profile of CTB has been demonstrated in numerous published studies in mice and non-human primates following systemic and intranasal dosing, as reviewed in Bauldauf et al. (37). In this study, TB-PCF showed no evidence of cytotoxicity or epithelial barrier disruption in an *in vitro* human alveolar epithelial model, consistent with CTB’s known tolerability. However, these results are confined to acute epithelial responses and observational readouts *in vivo*; potential systemic, neurotropic, or chronic inflammatory effects remain to be formally evaluated in dedicated toxicology studies.

Unlike viral pathogens, where a single antigen is often a sufficient target to induce protective immunity, Mtb is a complex bacterial pathogen, displaying over 4000 protein-coding genes (38). However, only a small number of these antigens have ever been tested in vaccines, and none has ever been shown to induce complete protection in experimental studies. Studies utilising ESAT6 and CFP10 have demonstrated that these early infection phase antigens are critical for inducing robust Th1-mediated immunity, particularly IFN-γ production, with the live-attenuated MTBVAC candidate which retains these antigens, exhibiting superior protection over BCG in macaques and mice by priming specific T cell responses absent in the BCG vaccine (39, 40). In animal models, subunit vaccines such as H1 (Ag85B-ESAT6) and H56 (adding Rv2660c) showed significant prophylactic and post-exposure protection, with ESAT6 being uniquely effective in preventing disease reactivation (41). Both candidates are currently in the clinical phase of testing. Other antigens commonly used in subunit TB vaccines are Ag85B (e.g., in H1, H4, H56), Ag85A (e.g., in MVA85A), TB10.4 (e.g. in H4 and AERAS-402 candidates), Rv1196 and Rv0125 (e.g. in M72 vaccine), Rv2660c (e.g. in H56), Rv1813 and Rv3619c (e.g. in ID93) (42). Therefore, the choice of antigen(s) is critical for subunit vaccine design, but unfortunately, the current tendencies are to base it on prior evidence rather than new antigen screening. This is one of the main roadblocks in TB vaccine testing and development, but while our choice of ESAT6-CFP10 is also based on prior evidence, our primary goal was to test this novel mucosal vaccine platform, that can be readily adapted for a variety of antigens. In addition to being prototype antigens to test our new vaccine platform, the choice of ESAT6-CFP10 was supported by the fact that these two secreted proteins naturally form heterodimers (43), which could translate into more physiologically relevant vaccine immune responses then when used individually. Our immunogenicity data revealed strong Th1 responses induced by TB-PCF, with immune splenocytes able to control bacterial infection *in vitro*, but unfortunately this failed to translate into protection *in vivo*. This outcome may stem from several factors, but we suspect that the choice of ESAT6-CFP10 is one contributor, as these two antigens may not provide sufficient antigenic breadth to confer complete protection. Therefore, additional or alternative antigens such as Ag85B, Rv2660c, or Mtb39A should be evaluated using the PCF platform. This approach can enable systematic antigen screening, not only to expand immune coverage but also to investigate how factors such as the kinetics of immune priming, the induction and persistence of lung-resident memory T cells (TRM), and the spatial organisation of mucosal immune responses collectively shape protective immunity against TB.

Finally, though TB-PCF construct can be expressed in mammalian expression systems, we opted for plant expression for several reasons. Plant-based expression of complex proteins such as TB-PCF offers a transformative “molecular farming” approach compared to traditional bioreactors [8]. This system leverages plants as low-cost bio-factories, enabling the rapid scale-up of antigen production through greenhouse expansion rather than capital-intensive fermentation infrastructure. Yields are bolstered by using viral-mediated expression in species such as *Nicotiana benthamiana*, which can produce complex proteins at a fraction of the cost of mammalian cell lines (44). Critically, for low- and middle-income countries (LMICs), plant systems could allow for local ownership of manufacturing, bypassing expensive cold-chain logistics and patent dependencies associated with global supply chains. This decentralisation ensures direct access to vaccines in regions with the highest TB burden.

In conclusion, systemic priming and mucosal boosting with the plant-produced TB-PCF vaccine successfully induced robust, polyfunctional Th1/Th17 responses and mucosal IgA. While the platform proved safe and immunologically active, with immune splenocytes able to kill infected macrophages *in vitro*, the limited *in vivo* protection suggests that ESAT6-CFP10 alone may not provide sufficient antigenic breadth. Nonetheless, TB-PCF represents a flexible new tool for screening more diverse antigen combinations to overcome current roadblocks in pulmonary TB vaccine development.

## Data Availability

The original contributions presented in the study are included in the article/**Supplementary Material**. Further inquiries can be directed to the corresponding author.

## Supplementary material

Supplementary Figure 1 The confidence level of prediction of TB-PCF by AlphaFold2. The pLDDT score ranges from 0 to 100: very high confidence in dark blue (pLDDT > 90); confident in light blue (90 > pLDDT > 70); low confidence in yellow (70 > pLDDT > 50); very low confidence in orange (pLDDT < 50). The accuracy is indicated with Color PAE (Predicted aligned errors).

Supplementary Figure 2 Matching of ESTA6-CFP10 structure between TB-PCF predicted by AlphaFold 2 and X-ray diffraction. **(a)** Monomeric TB-PCF matched to the X-ray 3D structure of the heterodimer of CFP10-ESAT6 complex from *Mtb*. The reference model is from the PDB 3FAV (21). ESAT6 in TB-PCF (light blue); ESAT6 ESXA in 3FAV (light pink); CFP10 in TB-PCF (violet); ESAT6-like protein ESXB in 3FAV (dark pink). Structural alignment using ChimeraX Matchmaker revealed a high degree of similarity between the best TB-PCF model and the ESAT6 subunit (EsxA), with an RMSD of 0.731 Å over 61 pruned atom pairs, indicating strong agreement in the conserved structural core. In addition, alignment with the CFP10 subunit (EsxB; ESAT6–like protein) showed near-identical structures, yielding an RMSD of 0.703 Å across all 74 aligned atom pairs. Together, these results confirm the high accuracy and structural reliability of the modeled TB-PCF structure. **(b)** The boxes show the sequences of ESAT6 and CFP10 within TB-PCF aligned to individual X-ray 3D structure of each protein, respectively.

## References

[1] World Health Organization . Global Tuberculosis Report 2024. Geneva: World Health Organization (2024).

[2] Colditz GA . Efficacy of BCG vaccine in the prevention of tuberculosis. JAMA. (1994) 271:698. doi: 10.1001/jama.1994.03510330076038 8309034

[3] Andersen P Doherty TM . The success and failure of BCG — implications for a novel tuberculosis vaccine. Nat Rev Microbiol. (2005) 3:656–62. doi: 10.1038/nrmicro1211 16012514

[4] De Gregorio E Tritto E Rappuoli R . Alum adjuvanticity: Unraveling a century old mystery. Eur J Immunol. (2008) 38:2068–71. doi: 10.1002/eji.200838648 18651701

[5] Xing J Zhao X Li X Fang R Sun M Zhang Y . The recent advances in vaccine adjuvants. Front Immunol. (2025) 16:1557415. doi: 10.3389/fimmu.2025.1557415 40433383 PMC12106398

[6] Lewis DJM Huo Z Barnett S Kromann I Giemza R Galiza E . Transient facial nerve paralysis (Bell’s palsy) following intranasal delivery of a genetically detoxified mutant of Escherichia coli heat labile toxin. PloS One. (2009) 4:e6999. doi: 10.1371/journal.pone.0006999 19756141 PMC2737308

[7] Baker K Rath T Lencer WI Fiebiger E Blumberg RS . Cross-presentation of IgG-containing immune complexes. Cell Mol Life Sci. (2013) 70:1319–34. doi: 10.1007/s00018-012-1100-8 22847331 PMC3609906

[8] Guilliams M Bruhns P Saeys Y Hammad H Lambrecht BN . The function of Fcγ receptors in dendritic cells and macrophages. Nat Rev Immunol. (2014) 14:94–108. doi: 10.1038/nri3582 24445665

[9] Kim MY Copland A Nayak K Chandele A Ahmed MS Zhang Q . Plant-expressed Fc-fusion protein tetravalent dengue vaccine with inherent adjuvant properties. Plant Biotechnol J. (2018) 16:1283–94. doi: 10.1111/pbi.12869 29223138 PMC5999314

[10] Kim MY Mason HS Ma JKC Reljic R . Recombinant immune complexes as vaccines against infectious diseases. Trends Biotechnol. (2024) 42:1427–38. doi: 10.1016/j.tibtech.2024.05.004 38825437

[11] Kim MY Reljic R Kilbourne J Ceballos-Olvera I Yang MS Reyes-del Valle J . Novel vaccination approach for dengue infection based on recombinant immune complex universal platform. Vaccine. (2015) 33:1830–8. doi: 10.1016/j.vaccine.2015.02.036 25728317

[12] Kim M Van Dolleweerd C Copland A Paul MJ Hofmann S Webster GR . Molecular engineering and plant expression of an immunoglobulin heavy chain scaffold for delivery of a dengue vaccine candidate. Plant Biotechnol J. (2017) 15:1590–601. doi: 10.1111/pbi.12741 28421694 PMC5698049

[13] Kim M Vergara E Tran A Paul MJ Kwon T Ma JKC . Marked enhancement of the immunogenicity of plant‐expressed IgG‐Fc fusion proteins by inclusion of cholera toxin non‐toxic B subunit within the single polypeptide. Plant Biotechnol J. (2024) 22:1402–16. doi: 10.1111/pbi.14275 38163285 PMC11022806

[14] Kim M Tran AC Kim J Ayuk HS Sparrow A Bossi L . Structural design and immunogenicity of a novel self‐adjuvanting mucosal vaccine candidate for SARS‐CoV‐2 expressed in plants. Plant Biotechnol J. (2025) 24:pbi.70278. doi: 10.1111/pbi.70278 40692205 PMC12854891

[15] Webster GR Van Dolleweerd C Guerra T Stelter S Hofmann S Kim M . A polymeric immunoglobulin—antigen fusion protein strategy for enhancing vaccine immunogenicity. Plant Biotechnol J. (2018) 16:1983–96. doi: 10.1111/pbi.12932 29682888 PMC6230950

[16] Merritt EA Sarfaty S Akker D L’Hoir C Martial JA Hol’ GJ . Crystal structure of cholera toxin B-pentamer bound to receptor GMl pentasaccharide. Protein Sci. (1994) 3:166–75. doi: 10.1002/pro.5560030202 PMC2142786 8003954

[17] Tran AC Boariu E García-Bengoa M Kim MY Vergara EJ Mussá T . Serological analysis reveals differential antibody responses between TB patients and latently infected individuals from the TB endemic country of Mozambique. Front Med. (2023) 10:1286785. doi: 10.3389/fmed.2023.1286785 37877025 PMC10591198

[18] Falero-Diaz G Challacombe S Rahman D Mistry M Douce G Dougan G . Transmission of IgA and IgG monoclonal antibodies to mucosal fluids following intranasal or parenteral delivery. Int Arch Allergy Immunol. (2000) 122:143–50. doi: 10.1159/000024370 10878493

[19] White AD Tran AC Sibley L Sarfas C Morrison AL Lawrence S . Spore-FP1 tuberculosis mucosal vaccine candidate is highly protective in Guinea pigs but fails to improve on BCG-conferred protection in non-human primates. Front Immunol. (2023) 14:1246826. doi: 10.3389/fimmu.2023.1246826 37881438 PMC10594996

[20] Vergara EJ Tran AC Paul MJ Harrison T Cooper A Reljic R . A modified mycobacterial growth inhibition assay for the functional assessment of vaccine-mediated immunity. NPJ Vaccines. (2024) 9:123. doi: 10.1038/s41541-024-00906-z 38956057 PMC11219912

[21] Poulsen C Panjikar S Holton SJ Wilmanns M Song YH . WXG100 protein superfamily consists of three subfamilies and exhibits an α-helical C-terminal conserved residue pattern. PloS One. (2014) 9:e89313. doi: 10.1371/journal.pone.0089313 24586681 PMC3935865

[22] Brosch R Gordon SV Garnier T Eiglmeier K Frigui W Valenti P . Genome plasticity of BCG and impact on vaccine efficacy. Proc Natl Acad Sci. (2007) 104:5596–601. doi: 10.1073/pnas.0700869104 17372194 PMC1838518

[23] Reljic R . IFN-γ therapy of tuberculosis and related infections. J Interferon Cytokine Res. (2007) 27:353–64. doi: 10.1089/jir.2006.0103 17523867

[24] Ogongo P Porterfield JZ Leslie A . Lung tissue resident memory T-cells in the immune response to Mycobacterium tuberculosis. Front Immunol. (2019) 10:992. doi: 10.3389/fimmu.2019.00992 31130965 PMC6510113

[25] Mangtani P Abubakar I Ariti C Beynon R Pimpin L Fine PEM . Protection by BCG vaccine against tuberculosis: A systematic review of randomized controlled trials. Clin Infect Dis. (2014) 58:470–80. doi: 10.1093/cid/cit790 24336911

[26] Martinez L Cords O Liu Q Acuna-Villaorduna C Bonnet M Fox GJ . Infant BCG vaccination and risk of pulmonary and extrapulmonary tuberculosis throughout the life course: a systematic review and individual participant data meta-analysis. Lancet Glob Health. (2022) 10:e1307–16. doi: 10.1016/S2214-109X(22)00283-2 35961354 PMC10406427

[27] Perdomo C Zedler U Kühl AA Lozza L Saikali P Sander LE . Mucosal BCG vaccination induces protective lung-resident memory T cell populations against tuberculosis. mBio. (2016) 7:e01686-16. doi: 10.1128/mBio.01686-16 27879332 PMC5120139

[28] Kaufmann SHE . Tuberculosis vaccines: Time to think about the next generation. Semin Immunol. (2013) 25:172–81. doi: 10.1016/j.smim.2013.04.006 23706597

[29] Cerutti A . The regulation of IgA class switching. Nat Rev Immunol. (2008) 8:421–34. doi: 10.1038/nri2322 18483500 PMC3062538

[30] Tran AC Diogo GR Paul MJ Copland A Hart P Mehta N . Mucosal therapy of multi-drug resistant tuberculosis with IgA and interferon-γ. Front Immunol. (2020) 11. doi: 10.3389/fimmu.2020.582833 33193394 PMC7606302

[31] Williams A Reljic R Naylor I Clark SO Falero-Diaz G Singh M . Passive protection with immunoglobulin A antibodies against tuberculous early infection of the lungs. Immunology. (2004) 111:328–33. doi: 10.1111/j.1365-2567.2004.01809.x 15009434 PMC1782424

[32] Darrah PA Zeppa JJ Maiello P Hackney JA Wadsworth MH Hughes TK . Prevention of tuberculosis in macaques after intravenous BCG immunization. Nature. (2020) 577:95–102. doi: 10.1038/s41586-019-1817-8 31894150 PMC7015856

[33] Dijkman K Sombroek CC Vervenne RAW Hofman SO Boot C Remarque EJ . Prevention of tuberculosis infection and disease by local BCG in repeatedly exposed rhesus macaques. Nat Med. (2019) 25:255–62. doi: 10.1038/s41591-018-0319-9 30664782

[34] Stylianou E Paul MJ Reljic R McShane H . Mucosal delivery of tuberculosis vaccines: a review of current approaches and challenges. Expert Rev Vaccines. (2019) 18:1271–84. doi: 10.1080/14760584.2019.1692657 31876199 PMC6961305

[35] Holmgren J Lönnroth I Månsson J Svennerholm L . Interaction of cholera toxin and membrane GM1 ganglioside of small intestine. Proc Natl Acad Sci. (1975) 72:2520–4. doi: 10.1073/pnas.72.7.2520 1058471 PMC432800

[36] Saif-Ur-Rahman K Mamun R Hasan M Meiring JE Khan MA . Oral killed cholera vaccines for preventing cholera. Cochrane Database Syst Rev. (2024) 1:CD014573. doi: 10.1002/14651858.CD014573 38197546 PMC10777452

[37] Baldauf K Royal J Hamorsky K Matoba N . Cholera toxin B: One subunit with many pharmaceutical applications. Toxins. (2015) 7:974–86. doi: 10.3390/toxins7030974 25802972 PMC4379537

[38] Cole ST Brosch R Parkhill J Garnier T Churcher C Harris D . Deciphering the biology of Mycobacterium tuberculosis from the complete genome sequence. Nature. (1998) 393:537–44. doi: 10.1038/31159 9634230

[39] Aguilo N Gonzalo-Asensio J Alvarez-Arguedas S Marinova D Gomez AB Uranga S . Reactogenicity to major tuberculosis antigens absent in BCG is linked to improved protection against Mycobacterium tuberculosis. Nat Commun. (2017) 8:16085. doi: 10.1038/ncomms16085 28706226 PMC5519979

[40] White AD Sibley L Sarfas C Morrison A Gullick J Clark S . MTBVAC vaccination protects rhesus macaques against aerosol challenge with M. tuberculosis and induces immune signatures analogous to those observed in clinical studies. NPJ Vaccines. (2021) 6:4. doi: 10.1038/s41541-020-00262-8 33397991 PMC7782851

[41] Aagaard C Hoang T Dietrich J Cardona PJ Izzo A Dolganov G . A multistage tuberculosis vaccine that confers efficient protection before and after exposure. Nat Med. (2011) 17:189–94. doi: 10.1038/nm.2285 21258338

[42] Xu B Yuan M Yang L Huang L Li J Tan Z . Recent advances in clinical research of prophylactic vaccines against tuberculosis. Vaccines. (2025) 13:959. doi: 10.3390/vaccines13090959 41012162 PMC12474261

[43] Renshaw PS Panagiotidou P Whelan A Gordon SV Hewinson RG Williamson RA . Conclusive evidence that the major T-cell antigens of the Mycobacterium tuberculosis complex ESAT-6 and CFP-10 form a tight, 1:1 complex and characterization of the structural properties of ESAT-6, CFP-10, and the ESAT-6·CFP-10 complex. J Biol Chem. (2002) 277:21598–603. doi: 10.1074/jbc.M201625200 11940590

[44] Rosales-Mendoza S Govea-Alonso DO . The potential of plants for the production and delivery of human papillomavirus vaccines. Expert Rev Vaccines. (2015) 14:1031–41. doi: 10.1586/14760584.2015.1037744 25882610

