# Supplementary figures and images for: Immunogenicity and protective potential of a mucosal protein-only vaccine candidate for tuberculosis
Source: Front Immunol. 2026 Jun 23;17:1810847. doi: 10.3389/fimmu.2026.1810847 (PMC13337862; doi:10.3389/fimmu.2026.1810847)

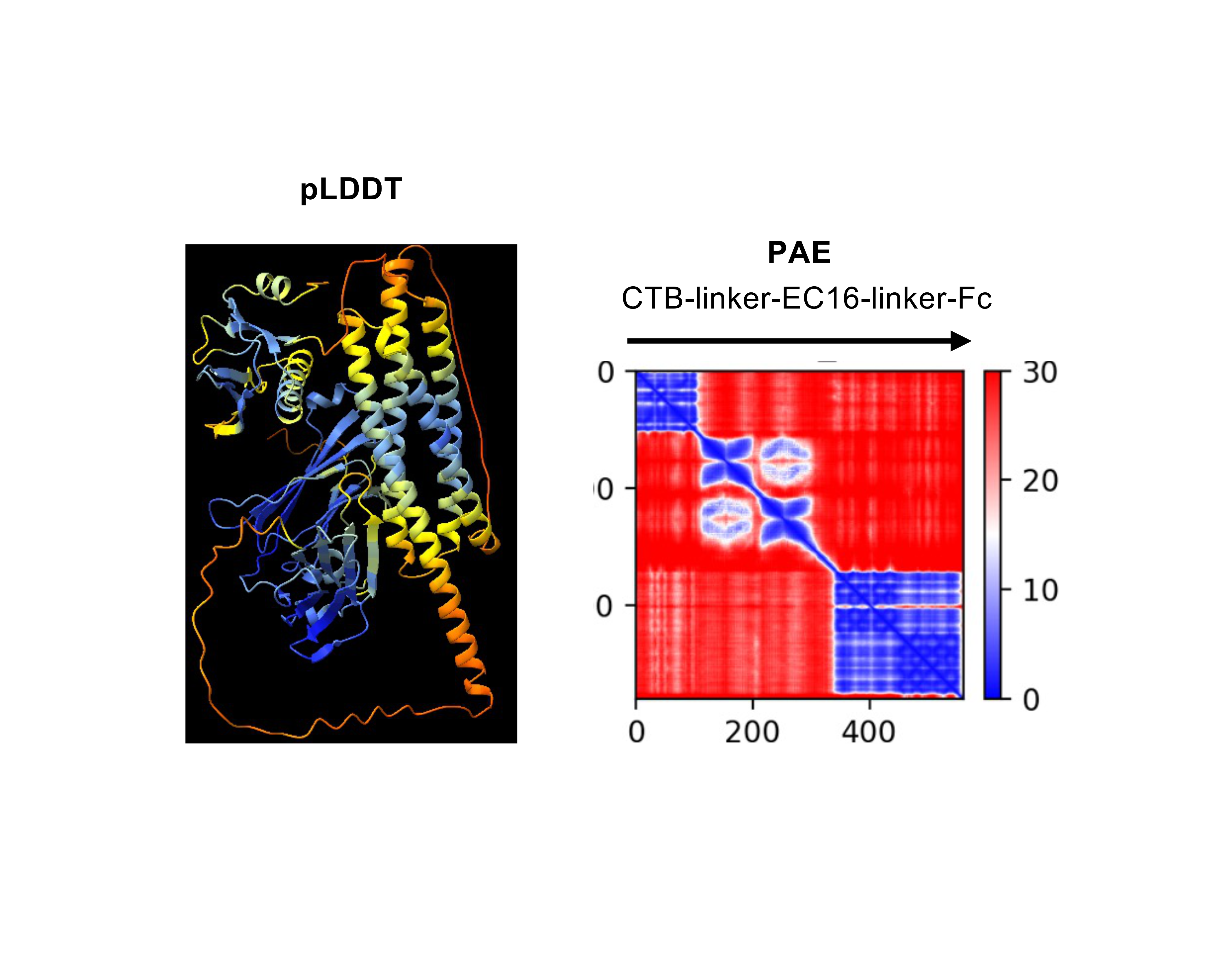

Supplement: Supplementary Figure 1 — The confidence level of prediction of TB-PCF by AlphaFold2. The pLDDT score ranges from 0 to 100: very high confidence in dark blue (pLDDT > 90); confident in light blue (90 > pLDDT > 70); low confidence in yellow (70 > pLDDT > 50); very low confidence in orange (pLDDT < 50). The accuracy is indicated with Color PAE (Predicted aligned errors). [file Image1.tiff]

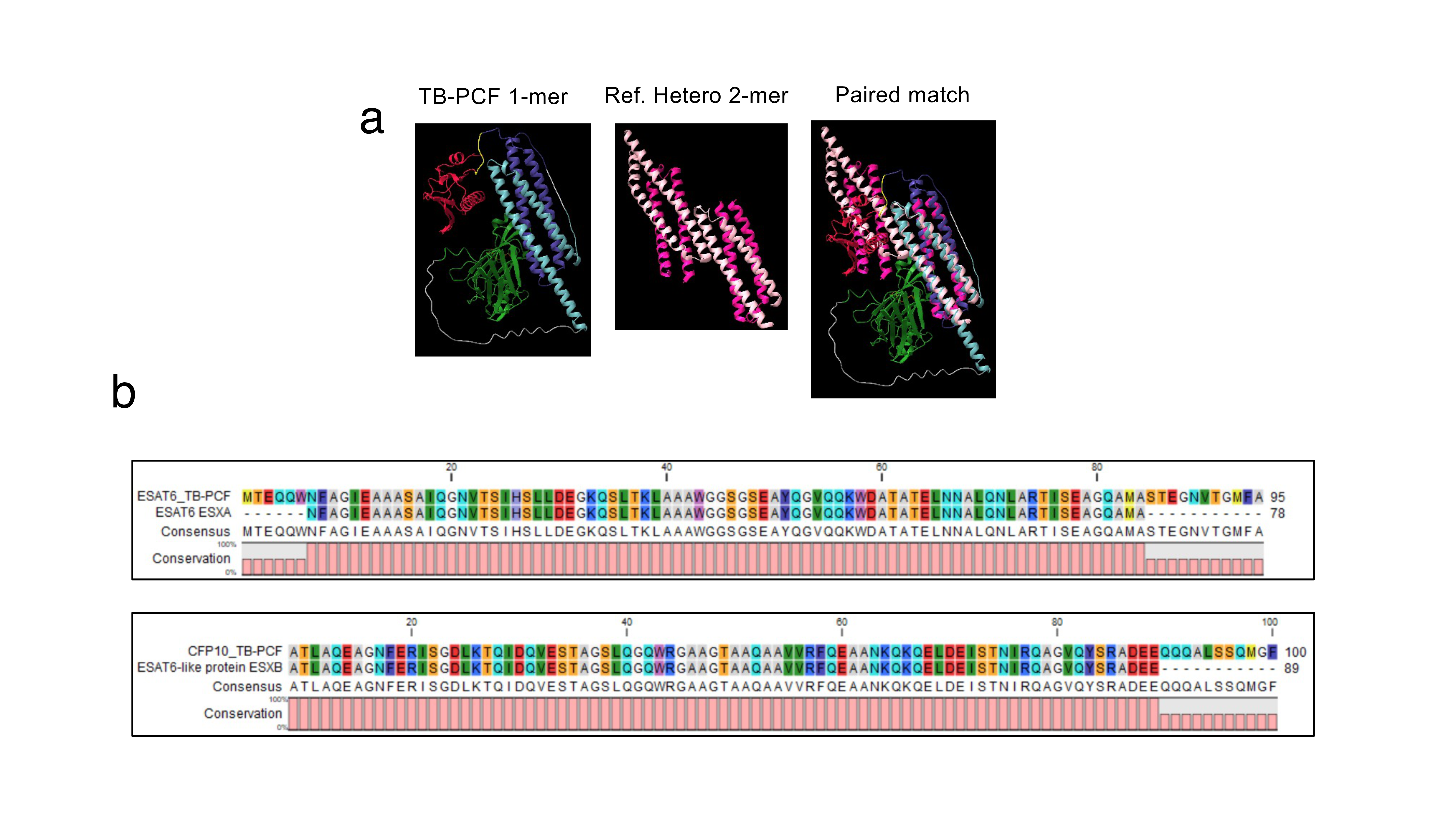

Supplement: Supplementary Figure 2 — Matching of ESTA6-CFP10 structure between TB-PCF predicted by AlphaFold 2 and X-ray diffraction. (a) Monomeric TB-PCF matched to the X-ray 3D structure of the heterodimer of CFP10-ESAT6 complex from Mtb. The reference model is from the PDB 3FAV (21). ESAT6 in TB-PCF (light blue); ESAT6 ESXA in 3FAV (light pink); CFP10 in TB-PCF (violet); ESAT6-like protein ESXB in 3FAV (dark pink). Structural alignment using ChimeraX Matchmaker revealed a high degree of similarity between the best TB-PCF model and the ESAT6 subunit (EsxA), with an RMSD of 0.731 Å over 61 pruned atom pairs, indicating strong agreement in the conserved structural core. In addition, alignment with the CFP10 subunit (EsxB; ESAT6–like protein) showed near-identical structures, yielding an RMSD of 0.703 Å across all 74 aligned atom pairs. Together, these results confirm the high accuracy and structural reliability of the modeled TB-PCF structure. (b) The boxes show the sequences of ESAT6 and CFP10 within TB-PCF aligned to individual X-ray 3D structure of each protein, respectively. [file Image2.tiff]
